# A combined treatment with selective androgen and estrogen receptor modulators prevents bone loss in orchiectomized rats

**DOI:** 10.1007/s40618-022-01865-9

**Published:** 2022-07-22

**Authors:** M. Komrakova, G. Büchler, K. O. Böker, W. Lehmann, A. F. Schilling, P. J. Roch, S. Taudien, D. B. Hoffmann, S. Sehmisch

**Affiliations:** 1 Department of Trauma Surgery, Orthopaedics and Plastic Surgery, University Medical Center Goettingen, Robert-Koch St. 40, 37075 Goettingen, Germany; 2 Department of Trauma Surgery, Hannover Medical School, University of Hannover, Carl-Neuberg-Str. 1, 30625 Hannover, Germany; 3 Division of Infection Control and Infectious Diseases, Georg-August-University of Goettingen, Humboldtallee 34A, 37073 Goettingen, Germany

**Keywords:** Enobosarm, Raloxifene, SARM, SERM, Male rats, Osteoporosis prophylaxis

## Abstract

**Purpose:**

Enobosarm (EN), a selective androgen receptor modulator and raloxifene (RAL), a selective estrogen receptor modulator, have been shown to improve bone tissue in osteoporotic males. The present study evaluated the effects of a combination therapy of EN and RAL on bone properties in orchiectomized rats compared to the respective single treatments.

**Methods:**

Eight-month-old male Sprague–Dawley rats were either left intact (Non-Orx) or orchiectomized (Orx). The Orx rats were divided into four groups (n = 15 each): 1) Orx, 2) EN treatment (Orx + EN), 3) RAL treatment (Orx + RAL), 4) combined treatment (Orx + EN + RAL). EN and RAL (0.4 mg and 7 mg/kg body weight/day) were applied immediately after Orx with a soy-free pelleted diet for up to 18 weeks. The lumbar spine and femora were examined by micro-CT, biomechanical, histomorphological, ashing, and gene expression analyses.

**Results:**

EN exhibited an anabolic effect on bone, improving some of its parameters in Orx rats, but did not affect biomechanical properties. RAL exhibited antiresorptive activity, maintaining the biomechanical and trabecular parameters of Orx rats at the levels of Non-Orx rats. EN + RAL exerted a stronger effect than the single treatments, improving most of the bone parameters. Liver weight increased after all treatments; the kidney, prostate, and levator ani muscle weights increased after EN and EN + RAL treatments. BW was reduced due to a decreased food intake in the Orx + RAL group and due a reduced visceral fat weight in the Orx + EN + RAL group.

**Conclusion:**

The EN + RAL treatment appeared to be promising in preventing male osteoporosis, but given the observed side effects on liver, kidney, and prostate weights, it requires further investigation.

**Supplementary Information:**

The online version contains supplementary material available at 10.1007/s40618-022-01865-9.

## Introduction

In men, hypogonadism and an age-related decline in gonadal hormone levels are associated with a decrease in bone mass, deterioration of bone structure, and the development of osteoporosis [1]. Furthermore, the pharmacological deprivation or surgical ablation of gonadal androgens applied to prostate-cancer patients can lead to the development of osteoporosis and osteoporosis-related fractures [2]. Men who sustain osteoporosis-related hip fractures have an increased mortality risk [3]. Published data have demonstrated bone structural changes with aging and, significantly, ongoing trabecular bone loss starting in young-adult men [4]. Despite increasing recognition of the problem of male osteoporosis, considerable gaps remain in the knowledge regarding this disorder and its treatment [3]. According to the guidelines on male bone diseases released by the European Academy of Andrology, the prevention or management of male osteoporosis can be performed by a testosterone replacement, though some additional anti-osteoporotic drugs may be needed in some cases [5].

Testosterone is an essential hormone for maintaining bone and muscle mass in men [5, 6]. A recent review and meta-analysis study demonstrated an inhibition of bone resorption and an increased bone mass in hypogonadal patients undergoing testosterone replacement therapy [7].

The skeletal actions of androgens may be partially mediated via estrogen receptors (ERs) after their conversion to estrogens by the action of aromatase [7]. Therefore, testosterone action on bone is dependent on the stimulation of both androgen receptors (ARs) and ERs [7, 8]. Moreover, it was shown that not only serum testosterone but also serum estradiol levels are important predictors of fracture risk in men [9]. Thus, potential use of testosterone as an osteoporosis treatment may require combining it with other bone active agents [5].

A previous clinical study demonstrated that estrogen therapy in combination with androgen therapy is more beneficial for body composition, muscle mass, and strength than estrogen therapy alone in postmenopausal women [10]. However, there are insufficient data for any conclusions regarding the efficacy and safety of testosterone-estrogen combination therapy in both genders.

The major concern for testosterone or estrogen replacement therapy is negative side effects [6, 11]. The application of non-steroidal selective androgen or estrogen receptor modulators (SARMs or SERMs) is gaining interest for treating osteoporosis and frailty, with the advantages of affecting musculoskeletal tissue selectively and causing fewer side effects than gonadal steroid-based hormones [12, 13].

Enobosarm (EN, Ostarine, MK-2866, or GTx-024) is a SARM that has been shown to increase muscle mass in patients with tumor cachexia and improve bone parameters in experimental studies [12, 14, 15]. Raloxifene (RAL) is a SERM that is an approved treatment for postmenopausal osteoporosis, which has been reported to affect bone turnover markers in elderly men and prevent bone loss in men with prostate cancer without feminizing effects [16, 17]. In our previous studies, we demonstrated the beneficial effects of RAL or EN applied as single treatments on bone and muscle tissue in an orchiectomized rat model [15, 18, 19]. Therefore, we hypothesized that the combination of EN and RAL would exert a favorable effect on bone tissue in male rats. The data on the combination of SERM and SARM in the literature are rare. Furuya et al. [20] reported positive additive effects of the application of a non-steroidal SARM (S-101479) combined with RAL on the bones of an ovariectomized rat model of postmenopausal osteoporosis. Our latest study showed improvement in muscle tissue under combined treatment of EN and RAL in male rats [19].

In the present study, we examined for the first time the effects of a combination treatment of EN and RAL on bone tissue and compared it to treatments with the respective single compounds in an orchiectomized rat model of male osteoporosis. The treatments were applied immediately after orchiectomy as an osteoporosis prophylaxis. Data on body weight, food intake and preliminary data of *in-vivo* computed tomography of the lumbar spine, as a part of this study, have been reported recently [19].

## Materials and methods

### General procedures

Seventy-five eight**-**month-old male Sprague–Dawley rats were obtained from Janvier (Le Genest-Saint-Isle, France) as retired breeders. The body weight averaged 692 ± 72 g among the rats. After 2 weeks acclimatization period, fifteen rats were left intact (non-orchiectomized, Group 1: Non-Orx), whereas sixty rats were orchiectomized (Orx) under isoflurane anesthesia. The Orx rats were divided into 4 groups, each with 15 rats: 1) untreated Orx rats (Orx), 2) Orx rats treated with EN (Orx + EN), 3) Orx rats treated with RAL (Orx + RAL), and 4) Orx rats treated with EN and RAL (Orx + EN + RAL). Immediately after OVX, EN and RAL were administered to the rats along with a soy-free pelleted diet (Ssniff special diet GmbH, Soest, Germany) for up to 18 weeks. The average daily doses were 0.4 mg/kg body weight (BW) for EN and 7 mg/kg BW for RAL (Table 1). The doses were chosen based on previous studies [15, 18]. The rats were housed in numbers of three or four in standard cages under 12-h light:12-h darkness regimes at a constant temperature of 22 ± 2 °C. All rats received the same soy-free diet (Ssniff special diet GmbH) and demineralized water throughout the experiment. The BW of rats and their food intake were recorded weekly.

**Table 1:** Food intake, weight of body (BW) and internal organs, serum parameters and gene expression in L6 of Non-Orx and Orx rats either treated with enobosarm (EN), raloxifene (RAL) or combined treatment (EN + RAL)

Parameters	Non-Orx		Orx		Orx + EN		Orx + RAL		Orx + EN + RAL	
	Mean	SD	Mean	SD	Mean	SD	Mean	SD	Mean	SD
Averaged food intake (g/rat/day)^2^	29.8	0.6	27.1	1.3	28.6	0.9	22.3^1^	1.2	25.7^a^	2.8
Averaged dose, EN/RAL (mg/kg BW/day)^2^					0.37	0.05	6.65	1.15	0.40/7.9	0.08/1.58
Weights (g)										
Averaged BW	691	31	647^a^	40	665	38	584^a,b,c^	35	562^a,b,c^	25
Heart	1.87	0.26	1.62^a^	0.19	1.91^b^	0.28	1.48^a,c^	0.16	1.47^ac^	0.17
Liver	21.2	1.2	16.7^a^	1.5	20.8^b^	2.1	16.5^a,c^	1.2	18.3^1^	1.0
Kidney	4.02	0.32	3.29^a^	0.30	4.48^b^	0.84	3.44^a,c^	0.30	4.14^b,d^	0.37
Spleen	1.52	0.35	1.33	0.21	1.40	0.26	1.11^a,c^	0.15	1.15^a,c^	0.24
Lung	2.67	0.24	2.67	0.40	2.76	0.30	2.24^a,b,c^	0.35	2.37^c^	0.29
Visceral fat	16.8	5.4	18.3	5.7	17.4	5.9	14.3	5.5	7.8^1^	2.8
Prostate^2^	1.27^1^	0.38	0.24	0.06	0.63^b,d^	0.22	0.22	0.10	0.46^d^	0.11
Levator ani muscle^2^	1.91	0.33	0.91^a^	0.11	2.00^b,d^	0.22	0.81^a^	0.12	1.87^b,d^	0.13
Serum										
AP (U/l)^2^	147	59	138	28	165	45	148	39	200^a,b^	52
OC (ng/ml)	121	36	125	46	86	33	90	37	41^a,b^	16
CTX-I (ng/ml)	7.1	1.3	7.7	1.7	6.4	1.7	5.0^b^	0.8	4.5^a,b^	1.4
Ca (mmol/l)^2^	2.2	0.2	2.0^a^	0.2	2.1	0.2	2.1	0.1	2.2b	0.2
P (mmol/l)^2^	2.0	0.2	1.6^a^	0.2	1.8	0.2	1.7	0.2	2.0^b,d^	0.2
LH (pg/ml)	389	73	417	59	469	113	489	79	458	40
FSH (ng/ml)	7.4	1.7	7.5	0.7	8.6	1.2	9.5^a^	1.3	9.4	1.8
Gene expression, L6										
Opg	1.06	0.37	1.09	0.29	0.94	0.29	2.76^1^	1.13	1.73^c^	0.42
Rankl	1.03	0.24	1.91^a^	0.64	1.35	0.56	2.41^ac^	0.93	1.31^d^	0.33
Opg/Rankl	1.04	0.30	0.62	0.27	0.79	0.36	1.22^b^	0.56	1.49^b,c^	0.81
Oc	1.02	0.20	2.86	1.33	3.53^a^	1.63	7.10^a,b,c^	4.92	5.69^a,b^	1.87
Alp	1.00	0.07	0.97	0.12	0.80	0.27	1.01	0.39	0.79	0.08
ER-α	1.02	0.18	3.53^a^	1.33	4.87^a,b^	1.33	6.84^1^	1.15	2.26^c^	0.94
ER-β	1.18	0.59	6.91	5.55	6.83	6.64	15.21^1^	13.58	4.93	2.89
AR	1.01	0.14	3.14^a^	1.36	4.39^a,b^	1.42	6.57^1^	1.39	1.96^c^	0.72

^1^Differs from all other groups, ^a^differs from Non-Orx, ^b^differs from Orx, ^c^differs from Orx + EN, ^d^differs from Orx + RAL (*p* < 0.05, Dunn’s test: spleen, kidney, and P; Tukey test: all other data)

^2^data were published in Roch et al.[19]

After 18-week treatments, blood was collected using the cardiac puncture method under deep isoflurane anesthesia. Further, serum was stored at − 20 °C until analyses. The weights of the whole body (BW), visceral fat, heart, liver, kidney, spleen, lung, prostate, and levator ani muscle were recorded. The 4th lumbar vertebral bodies (L4) and left femora were dissected free of soft tissue and cartilage and stored at − 20 °C for further biomechanical, micro-computed tomographical (micro-CT), and ashing analyses. L5 was immersed in 4% buffered formalin for histomorphological analysis, and L6 was frozen in liquid nitrogen and then stored at − 80 °C for gene expression analysis.

### Serum analyses

Analyses of alkaline phosphatase (AP), calcium (Ca), magnesium (Mg), and phosphor (P) were conducted at the Department of Clinical Chemistry, University Medical Center, Goettingen using commercial tests (Architect, Abbott, Wiesbaden, Germany) and an automated chemistry analyzer (Architect c16000 Analyzer, Abbott). Osteocalcin (OC) and the cross-linked C-telopeptide of type-I collagen (CTX-I) were assessed using an enzyme-linked immunosorbent assay (EIA), rat-MID™ Osteocalcin EIA, and RatLaps (CTX-I) EIA, respectively (Immunodiagnostic Systems GmbH, Frankfurt am Main, Germany). Follicle stimulating hormone (FSH) and luteinizing hormone (LH) were measured by EIA kit for rats (Cloud-Clone Corp., Katy, Texas, USA).

### Micro-CT analyses

The left femur and L4 were scanned using a Quantum FX micro-CT (Caliper Sciences, Hopkinton, MA, USA) under the following scan protocol: 70 Kilovoltage peak (kVp), 200 μA, 2-min exposure time, 360° rotation, 3600 projections, 20 × 20 mm^2^ field-of-view, 512-pixel matrix, and 40 × 40 × 40 μm^3^ effective voxel size. Five hydroxyapatite elements of varying mineral densities (0.2, 0.4, 0.6, 0.8, and 1.0 g/cm^3^) were scanned with each bone to convert the data into bone mineral density (Suppl. Fig. S1). The scans were analyzed using a program (3D OsteoAnalyze) developed in our laboratory. A later version of this program is Scry v6.0 software (Kuchel and Sautter UG, Bad Teinach-Zavelstein, Germany) [21].

The region of interest in the femur was the femoral head, which was digitally cut in the transition zone from the collum femoris to the trochanter major (Suppl. Fig. S1). In L4, the corpus vertebra was separated (Suppl. Fig. S1). Standard thresholds for soft tissue, trabecular and cortical bone, bone tissue, and total tissue were found for the femur and L4 by averaging six measurements of visually detected thresholds (three samples each from Non-Orx and Orx groups) and used for all samples (Suppl. Fig. S1) [22]. The following three-dimensional (3D) bone parameters were measured according to ASBMR criteria: total tissue volume and density (Tt.V and Tt.BMD), trabecular volume and density (Tb.V and Tb.BMD), cortical volume and density (Ct.V and Ct.BMD), soft tissue volume and density (St.V and St.BMD), and bone volume fraction (BV/TV) [23].

Further structural analysis was performed using two-dimensional (2D) images created by the 3D OsteoAnalyze program (Suppl. Fig. S1). Three images of sagittal-cut femoral head and vertebral body were analyzed using MetaMorph Basic Acquisition Software (Leica Mikrosysteme Vertrieb GmbH, Wetzlar, Germany). The following parameters were measured: cortical density (Ct.Dn, % of bone tissue), trabecular density (Tb.Dn, %), number of trabecular nodes (N.Nd), and trabecular thickness (Tb.Wi, µm) [23, 24].

### Biomechanical analyses

The biomechanical properties of the left femora and L4 were analyzed using a Zwick testing machine (Zwick/Roell, type 145,660 Z020/TND, Ulm, Germany). A three-point bending test was performed on the femurs, which were placed on an aluminum base and loaded to the trochanteric region until broken (Suppl. Fig. S1). L4 was fixed at the aluminum base, and a stamp was loaded to the vertebral body, applying compression test (Suppl. Fig. S1). The stamp was loaded at 50 mm/min and stopped automatically by software (TestExpert, Zwick/Roell) when the applied force decreased more than 20 N for femurs and 10 N for L4. Stiffness (N/mm), the slope of the linear increase of the curve during elastic deformation, and the maximal force (Fmax, N) that the bone could withstand before it broke were assessed [22, 25, 26]. The parameters were calculated using Excel (Microsoft Office 2016).

### Ashing analyses

The left femur and L4 were ashed in a muffle oven at 750 °C for 2 h. The bones were weighed before and after ashing to the nearest 0.000001 g. Mineral content was determined by the ash weight. Organic content was calculated as the difference between the wet tissue weight and the ash weight. Organic content and mineral content were expressed relative to the wet weight of each bone (%) [27]. Calcium and magnesium content was assessed using an atomic absorption spectrometer (4100, PerkinElmer, Germany) according to the european committee for standardization (CEN) [28]. Orthophosphate content was determined using the colometric method (2030 Multilabel Reader Viktor X4, Perkin Elmer, Turku Finnland) according to CEN [29].

### Histomorphometrical analysis

L5 was fixed in 4% buffered formalin for 1 week and then stored for a few weeks in 70% ethanol. Thereafter, L5 was embedded in Technovit 9100 New® (Heraeus Kulzer GmbH, Wehrheim, Germany) and cut longitudinally using a Leica microtome (RM 2165, Leica Instruments GmbH) to a thickness of 5 µm. The sections were deacrylated, stained with Toluidine Blue O (Merck, Darmstadt, Germany), and mounted with Eukitt (O. Kindler GmbH, Freiburg, Germany) [30]. The sections were digitalized using a digital camera (Leica DFC490) and a zoom stereo microscope (Leica DMRXE) and analyzed with the aid of the MetaMorph image analysis program (Leica, Bensheim, Germany). Three randomly chosen fields of 0.1 mm^2^ within the histological section were taken for the analyses. The following parameters were measured according to ASBMR nomenclature: osteoblast number per bone perimeter (N.Ob/B.Pm), osteoclast number per B.Pm (N.Oc/B.Pm), and osteocyte number per bone area (Ot/B.Ar) [24, 31]. The criteria for the morphological identification of osteoblasts and osteoclasts were as follows. Cuboid-shaped cells that covered trabecular bone were counted as osteoblasts, whereas multinucleated cells that were resorbing bone were counted as active osteoclasts [32].

### Gene expression analyses

L6 was homogenized using a Mikro-Dismembrator S (Sartorius, Goettingen, Germany). Total cellular RNA was extracted using the RNeasy™ Mini Kit (Qiagen, Hilden, Germany), and 1000 ng of each RNA sample was reverse-transcribed using Superscript™ RNase H-reverse transcriptase (Promega, Mannheim, Germany). Gene expression analysis was done with the quantitative real-time polymerase chain reaction based on SYBR-Green detection (PCR QuantiTect® Sybr® Green Kit, Qiagen) using iCycler (CFX96, Bio-Rad Laboratories, Munich, Germany). Ready-to-use primer pairs were obtained from Qiagen (QuantiTect® Primer Assays). The expression of the following genes was analyzed: alkaline phosphatase (Alp), osteocalcin (Oc), the receptor activator of nuclear factor B ligand (Rankl), osteoprotegerin (Opg), estrogen receptor alpha (ER-α), estrogen receptor beta (ER-β), androgen receptor (AR), and reference gene β-2 microglobulin. The relative gene expression was calculated using the 2^−ΔΔCT^ method [33] relative to the Non-Orx group. The ratio of Opg to Rankl was calculated using Excel [34].

### Statistical analysis

Statistical analysis was performed using GraphPad Prism (Version 5.04, GraphPad Software, Inc. San Diego, USA). Gaussian distribution was tested for each parameter within the experimental group by applying Kolmogorov–Smirnov test, P´Agostino and Person omnibus test and Shapiro–Wilk test. The data were considered normally distributed if they passed at least one of the normality tests. Thereafter, for normally distributed variables, one-way ANOVA and Tukey-test were applied (*p* < 0.05). Nonparametric Kruskal–Wallis test and Dunn multiple comparison test were used for analysis of the variables which were not normally distributed (*p* < 0.05). Data are shown as medians and the interquartile ranges in box plot figures and as means (Mean) and standard deviations (SD) in tables.

## Results

### Animal model

The mean BW of the Orx + RAL and Orx + EN + RAL rats was significantly lower than that of the Non + Orx, Orx, and Orx + EN groups (Table 1). The food intake of the Orx + RAL rats was the lowest among the treatment groups, and that of the Orx + EN + RAL group was lower than that of Non-Orx group (Table 1). The average doses of the tested substances are shown in Table 1.

**Fig. 1:**
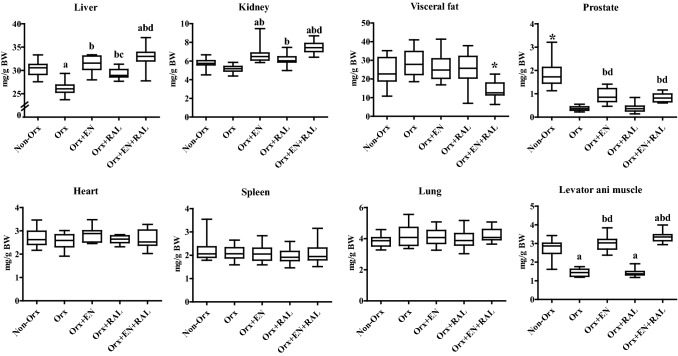
The relative weight of internal organs, visceral fat, prostata and levator ani shown in mg/ g body weight (BW). (*) differs from all other groups, **a** differs from Non-Orx, **b** differs from Orx, **c** differs from Orx + EN, **d** differs from Orx + RAL (*P* < 0.05, Dunn’s test: spleen, and kidney; Tukey test: all other data)

Orx caused a reduction in the absolute weights of the heart, liver, kidney, prostate, and levator ani muscle, whereas EN treatment enhanced these parameters (Table 1). The RAL treatment did not change most of these parameters, solely reducing the lung weight. The EN + RAL treatment increased the liver, kidney, prostate, and levator ani muscle weights and decreased the visceral fat weight. The weights of most organs, including the spleen, were lower in the Orx + RAL and Orx + EN + RAL groups than in the Non-Orx group (Table 1). A strong, positive correlation of the prostate, internal-organ, and visceral fat weights with BW was seen, as shown in Table 2. The correction of organ weights by BW revealed the enhanced liver weight in the Orx + EN, Orx + RAL, and Orx + EN + RAL groups, increased kidney, prostate, and levator ani muscle weights in the Orx + EN and Orx + EN + RAL groups, and decreased fat weight in the Orx + EN + RAL group (Fig. 1). The levator ani weight was not correlated with BW, and the correction of this by BW did not change the differences as shown in Table 1 and 2, and Fig. 1.

**Table 2:** Correlations of weight (W) of internal organs, prostate and levator ani with body weight (BW) assessed by Pearson´s coefficient (*r*), two-tailed *P*-value

Correlations (*n* = 68)	Pearson, *r*	*P*
Heart W–BW	0.642	< 0.0001
Liver W–BW	0.687	< 0.0001
Kidney W–BW	0.369	0.002
Spleen W–BW	0.574	< 0.0001
Lung W–BW	0.502	< 0.0001
Visceral fat W–BW	0.639	< 0.0001
Prostate W–BW	0.458	< 0.0001
Levator ani W–BW	0.057	0.647

### Serum analyses

The EN treatment did not change any of the serum parameters (Table 1). The RAL treatment decreased CTX-I levels and increased FSH levels. The EN + RAL treatment increased AP, Ca, and P levels and decreased OC and CTX-I levels. The Orx reduced Ca and P levels. The Mg level in serum did not differ between groups (P > 0.05), with an average of 0.7 ± 0.1 mmol/L [19].

### Micro-CT

**Fig. 2:**
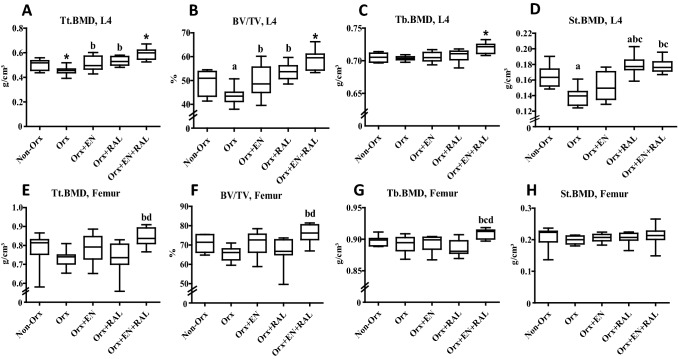
Micro-CT 3D analysis of L4 (**A**–**D**) and femur (**E**–**H**). Tt.BMD: total bone mineral density, BV/TV: bone volume fraction, Tb.BMD: trabecular BMD, St.BMD: soft tissue BMD. (*) differs from all other groups, **a** differs from Non-Orx, **b** differs from Orx, **c** differs from Orx + EN, (d) differs from Orx + RAL RAL (P < 0.05, Dunn’s test: BV/TV Femur, and St.BMD; Tukey test: all other data). (*P* < 0.05, Tukey-test).

A 3D analysis of L4 revealed that in the Orx + EN group, most of the bone volumetric parameters (BV/TV, Tb. V, and Ct.V) and Tt.BMD were enhanced compared with the Orx group (Table 3, Fig. 2A, B). In femur samples, St.V was lower in the Orx + EN group. The RAL treatment caused increases in Tt.BMD, Ct.BMD, St. BMD, BV/TV, and Tb.V, whereas Ct.BMD and St.V were reduced in L4 compared with Orx rats (Table 3, Fig. 2A, B, D). The combined EN + RAL treatment improved almost all bone parameters in L4, with the exception of Ct. BMD (Table 3, Fig. 2A–D). Similarly, in femur samples, most of the bone parameters were improved by this treatment; only St.BMD, Tb.V, and Ct.BMD were not changed (Table 3, Fig. 2E–H). Orx significantly impaired Tt.BMD, BV/TV, St.BMD, and Tb.V in L4 (Table 3, Fig. 2A, B, D). In femur samples, the bone parameters were also diminished in the Orx group, but this did not reach a significant level (Table 3, Fig. 2E–H).

**Table 3:** micro-CT, ashing and histomorphometrical analyses of lumbar vertebral body (L) and femur in Non-Orx and Orx rats either treated with enobosarm (EN), raloxifene (RAL) or combined treatment (EN + RAL)

Parameters	Non-Orx		Orx		Orx + EN		Orx + RAL		Orx + EN + RAL	
	Mean	SD	Mean	SD	Mean	SD	Mean	SD	Mean	SD
Micro-CT 3-D										
L4										
B.BMD (g/cm^3^)	0.85	0.02	0.86	0.02	0.88^d^	0.04	0.84	0.03	0.88^d^	0.04
BV (mm^3^)	64	7	49^1^	5	59	8	59	7	70^c,d^	7
Ct.BMD (g/cm3)	1.11	0.01	1.12	0.01	1.13	0.03	1.09^a,b,c^	0.02	1.10^c^	0.02
Ct.V (mm^3^)	23	3	19	3	24^b^	4	20	4	29^1^	6
Tb.V (mm^3^)	41	5	30^1^	3	35	6	39	6	41	5
St.V (mm^3^)	65	11	63	7	60	11	51^a,b^	7	49^a,b,c^	7
Femur										
B.BMD (g/cm^3^)	1.04	0.04	1.00	0.03	1.02	0.04	0.99^a^	0.03	1.05^b,d^	0.04
BV (mm^3^)	36	4	33	3	33	4	34	5	39^b^	5
Ct.BMD (g/cm^3^)	1.25	0.04	1.23	0.03	1.25	0.03	1.25	0.03	1.26	0.03
Ct.V (mm^3^)	12	3	10	2	12	3	10	3	15^bd^	4
Tb.V (mm^3^)	20	4	22	2	22	2	24	4	23	4
St.V (mm^3^)	14	3	17	2	14^b^	3	16	2	12^b,d^	2
Ashing analysis										
L4										
Anorganic content (%)	28.4	1.5	25.4^a^	3.5	27.3	2.6	28.0	3.0	30.0^b^	2.1
Organic content (%)	71.6	1.5	74.6^a^	3.5	72.7	2.6	72.2	3.0	70.3^b^	2.1
Mg^+^ (%)	0.68	0.02	0.66	0.01	0.70^b,d^	0.02	0.66	0.02	0.67	0.02
Ca^2+^/PO_4_^3−^	1.54	0.04	1.51	0.03	1.62^b^	0.13	1.71^a,b^	0.04	1.72^a,b,c^	0.03
Femur										
Anorganic content (%)	43.3	2.4	40.3^a^	2.1	43.9^b^	2.0	43.8^b^	2.1	47.3^1^	3.2
Organic content (%)	56.8	2.4	60.0^a^	2.1	56.1^b^	2.0	56.3^b^	2.1	52.7^1^	3.2
Mg^+^ (%)	0.66	0.02	0.66	0.01	0.67	0.01	0.65	0.02	0.65	0.02
Ca^2+^/PO_4_^3−^	1.26	0.05	1.25	0.04	1.25	0.03	1.25	0.04	1.29	0.05
Histomorphometry, L5										
Ob/B.Pm (N/mm)	57	13	60	12	71	14	69	15	66	23
Oc/B.Pm (N/mm)	2.8	0.6	3.3	0.9	3.2	1.3	2.8	1.0	2.8	0.9
Ot/B.Ar (N/mm^2^)	565	159	589	176	585	85	536	201	510	89

^1^differs from all other groups, a-differs from Non-Ovx, b- differs from Ovx, d- differs from OS-0.4 (*p* < 0.05, Dunn’s test: Femur BV, Femur Ct.BMD, Femur St.V, Ob/B.Pm, and Oc/B.Pm; Tukey test: all other data)

*B* Bone, *Ct* cortical bone, *Tb* trabecular bone, *St* soft tissue, *BMD* bone mineral density, *V* volume

**Fig. 3.**
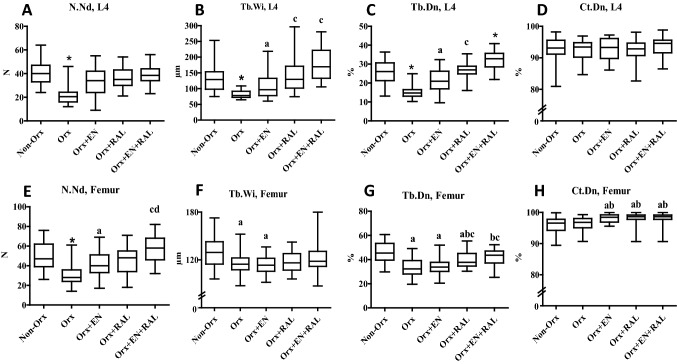
2D analysis of L4 (**A**–**D**) and femur (**E**–**H**). N.Nd: number of trabecular nodes, Tb.Wi: trabecular width, Tb.Dn: trabecular density, Ct.Dn: cortical density. (*) differs from all other groups, **a** differs from Non-Orx, **b** differs from Orx, **c** differs from Orx + EN, **d** differs from Orx + RAL RAL (*P* < 0.05, Dunn’s test: Tb.Wi L4, Tb.Dn L4, Tb.Wi Femur, and Ct.Dn. Femur; Tukey test: all other data). (*P* < 0.05, Tukey-test).

A 2D bone-structure analysis showed that the trabecular parameters were affected by all treatments in both L4 and femurs (Fig. 3A–C, E–G). The EN treatment improved the trabecular structure. The effect of RAL was stronger on bone than that of EN, whereas EN + RAL showed the strongest effect. Cortical density was not changed in L4, while it improved for femurs after EN, RAL, and EN + RAL treatments (Fig. 3D, H). Orx affected all trabecular parameters negatively, whereas cortical density was not changed (Fig. 3A–H).

### Biomechanical analyses

**Fig. 4:**
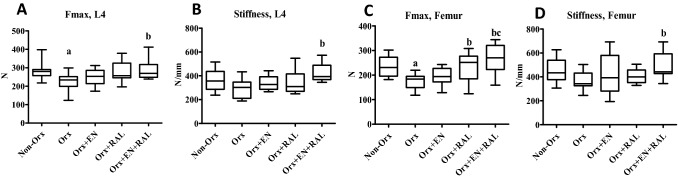
Biomechanical analysis of L4 (**A**, **B**) and femur (**C**, **D**). **a** differs from Non-Orx, **b** differs from Orx, **c** differs from Orx + EN (*P* < 0.05, Tukey test)

The EN treatment did not change the biomechanical parameters of femora or L4 (Fig. 4A-D), whereas RAL increased Fmax in femora. In the Orx + EN + RAL group, all parameters were higher than in the Orx groups. Orx significantly reduced the Fmax of L4 and femora (Fig. 4A, C).

### Histomorphometrical analysis

There were no differences (*P* > 0.05) in the Ob/B.Pm, Oc/B.Pm, and Ot/B.Ar of L5 between the treatment groups (Table 3).

### Ashing analyses

The anorganic content in femora was higher after EN and RAL treatments compared with the Orx group, whereas EN + RAL showed the highest anorganic content among all treatment groups (Table 3). The anorganic content of L4 in the Orx + EN and Orx + RAL groups did not differ from other groups, whereas EN + RAL treatment enhanced it (Table 2). In the Orx group, the anorganic content was significantly lower in both femora and L4 than in the Non-Orx group. The organic content corresponded to the changes observed for anorganic content, namely, increasing in the Orx group and decreasing in the other groups (Table 3). The femur Mg content was higher in the Orx + EN group than in the Orx group (Table 2), while in L4, there was no difference between groups.

### Gene expression analyses

In L6 of the Orx + EN group, the expression of ER-α and AR was higher than in the Non-Orx and Orx groups, and Oc expression was higher than in the Non-Orx group (Table 1). The Orx + RAL treatment increased the expression of most of the studied genes, as well as the Opg/Rankl ratio (Table 1). The EN + RAL treatment maintained the enhanced OC gene level and Opg/Rankl ratio, whereas the expression of Opg, Rankl, ER-α, ER-β, and AR diminished to the level of Non-Orx and Orx rats. Orx caused the upregulation of Rankl, ER-α, and AR expression compared to Non-Orx rats. The expression of Alp did not differ between the groups (Table 1).

## Discussion

In the present study, we showed that the combination of EN and RAL exerted a favorable effect on the bone tissue of Orx rats. The effect was stronger than that of single compounds. Most bone parameters were maintained or improved under the combined EN + RAL treatment. Treatment with RAL alone prevented bone deterioration, maintaining the biomechanical and trabecular parameters of Orx rats at the level of Non-Orx rats. Treatment with EN alone also affected bone tissue, improving some of the bone parameters in the Orx animals.

The EN effect on bone was seen in both in L4 and femora; however, it varied depending on the skeletal site. While cortical and trabecular volume, bone volume fraction, and structural parameters, Mg content, and Ca^2+^/PO_4_^3−^ ratio were enhanced in the L4 of the Orx + EN group, in femora, Ct.Dn and anorganic content were increased, and St.V and organic content were decreased. The degree and timing of changes in bone after aging, ovariectomy, or treatments were previously reported to differ between skeletal sites [35]. Most likely, the higher proportion of trabecular bone, which is metabolically more active than cortical bone [36], in L4 than in femora, could explain the more pronounced anabolic effect of EN on L4. The osteo-anabolic effect of SARMs through ARs has been reported previously [12–14] and the significantly higher mRNA expression of AR in L6 after EN treatment in our study confirmed this. ER-α was also enhanced in the present study after EN treatment, whereas ER-β expression was not changed. Both ERα and ERβ have been shown to correlate with bone mass [37]. ER-α and ER-β can partially compensate for each other, and also ER-β antagonizes many effects of ER-α [38]. The enhanced Ca^2+^/PO_4_^3−^ ratio in L4 and anorganic content in femur confirmed a favorable effect of EN on bone properties. Despite these changes in bone parameters, the EN treatment was not sufficient for reducing the progression of osteoporosis in Orx rats and maintaining biomechanical bone properties. Furthermore, the enhanced Mg content under EN treatment in L4 should be further investigated, since, despite evidence showing that Mg is beneficial to the skeleton, the elevated Mg levels might have harmful effects on bone metabolism and mineralization [39].

Similar to the EN effect, the RAL effect varied among skeletal sites. In L4, Tt.BMD, BV/TV, and Ca^2+^/PO_4_^3−^ increased, whereas Ct.BMD and St.V decreased. In femoral head, the 3D micro-CT parameters were not changed, whereas structural 2D parameters were improved under RAL treatment. In the entire femur, ashing analysis detected an enhanced anorganic content and Ca^2+^/PO_4_^3−^ ratio, as well as a diminished organic content. The osteoprotective effect of RAL was demonstrated by improved biomechanical properties of femur in this experiment. RAL exerts a selective estrogenic effect on bone via binding the ER-α and ER-β receptors, decreasing osteoclast differentiation and activity while maintaining the physiological function of osteoblasts at the same time [40]. Indeed, both ERs were expressed at a higher level in the RAL group than in the other groups. The expression of AR was also elevated in our study. The AR mRNA expression was shown to be upregulated by estrogen during the early postnatal period in male rat forebrain [41] (McAbee and DonCarlos 1999). An enhanced Opg expression and Opg/Rankl ratio in L4 of RAL-treated rats indicated that RAL modulated bone remodeling. RAL was reported to regulate the OPG/RANKL/RANK system in rats by increasing OPG levels and reducing RANKL and RANK expression [42].

The combined EN + RAL treatment showed a stronger effect on bone than the single treatments, where most of the osseous bone parameters (Tt.BMD, BV/TV, Tb.BMD, trabecular structure, and anorganic content) were enhanced, whereas St.V and organic content were reduced. The effect was seen similarly in both L4 and femur. Not only bone loss due to the sex hormone deficiency was prevented in Orx rats by this treatment, but also age-related bone loss was ameliorated when compared with Non-Orx rats, and the effect was strong enough to improve the biomechanical properties of bone. The elevated content of Mg under EN treatment was normalized, while the Opg/Rankl ratio, Oc expression, and Ca^2+^/PO_4_^3−^ ratio in L4 were at high levels, similar to those measured for RAL treatment. The elevated expression of ER-α, ER-β, and AR in the EN and RAL groups was reduced the level observed in Non-Orx and Orx rats. Antiresorptives such as RAL limit osteoclast activity and thus stabilize the mineralization rate of bone [40]. Osteo-anabolic SARMs such as EN are able to stimulate osteoblasts, which increases the mineralization rate [43]. Our results show the effectiveness of combining antiresorptive and osteo-anabolic therapies in preventing deterioration of bone tissue in the Orx rat model. Similarly, another SARM (S-101479) applied in combination with RAL improved bone parameters to the greater extend than single compounds in estrogen-deficient female rats [20]. In several clinical trials, bone anabolic treatment with teriparatide (PTH) was combined with antiresorptive medications [44]. The combination therapy of PTH and RAL increased bone formation compared to PTH alone; further bone resorption was reduced, enhancing the bone-forming effects of PTH [45]. The combination of PTH and bisphosphonate did not show substantial clinical benefits compared to the monotherapies [42], whereas the combination of PTH and denosumab demonstrated promising results [46].

In the present study, markers of bone turnover in serum were not affected by EN, whereas the RAL and EN + RAL treatments decreased CTX-I levels, with the latter also increasing AP activity and decreasing OC levels. This confirmed the antiresoptive activity of RAL [40] and osteo-anabolic effect of EN [41] applied with RAL in the Orx rats. Reduced OC levels after EN treatment were also detected in our previous study in Orx rats, which was explained by its possible antiresorptive activity [15], since not only the induction of osteoblast differentiation but also the inhibition of osteoclast differentiation were reported in previous *in-vitro* studies [47]. Most likely, the combination therapy of EN with RAL had an additive antiresorptive effect. Furthermore, the reduced Ca and P levels in the serum of Orx rats was restored by EN + RAL treatment, which confirms its favorable effect on bone tissue. This decrease in serum Ca and P levels is a known effect observed after Orx in rats [48]. We failed to detect differences between the groups at the cellular level as well as in serum markers of bone formation and resorption between Non-Orx and Orx groups after 18-week treatments in our study, perhaps because a transient increase in bone remodeling occurs earlier, one month post-Orx, whereas trabecular bone loss is observed four months after Orx [49, 50].

The low BW of Orx rats in our study is a known phenomenon that is observed after Orx in rats, which is independent of food intake [15, 51]. EN exerted no effect on BW, whereas RAL treatment caused a further reduction of BW due to the decreased food intake, which was also reported for RAL-treated ovariectomized rats [52]. In females, RAL has been shown to further reduce fat mass [52], which is in contrast to our study, as the visceral fat weight of males did not change after RAL treatment. Sex-related differences in the response of muscle tissue and fat deposition to RAL treatment have been described previously [53]. The combined treatment of RAL and EN also reduced BW. However, this was not due to a decreased food intake, as seen for RAL treatment alone, but due to the reduced visceral fat weight. Treatments with EN in Phase-I and Phase-II clinical trials demonstrated increased total lean body mass, enhancing functional performance, and decreased total tissue percent fat [54]. In our study, only the combined EN + RAL treatment effectively reduced fat weight.

Analyzing the weight of the internal organs we revealed its strong, positive correlation with BW, which is also seen in the literature [55]. The correction of data by BW showed higher liver and kidney weights in EN-treated rats than those in the Orx group. In a 12-week double-blind Phase-II trial, no increased rate of adverse effects was reported in an EN-treated group compared with a placebo group [56]. However, a recent case report described significant drug-induced liver injury attributed to the use of EN, similar to that associated with androgenic–anabolic steroids (AASs) [57]. RAL alone increased liver weight to a lesser extent than EN, which was significantly higher compared to the Orx group, but still lower than in the EN group. EN + RAL treatment elevated the liver weight compared to the Non-Orx, Orx, and RAL groups, but it remained at EN-group levels. In ovariectomized rats, it was shown that the pro-oxidant effect of RAL can perturb important liver metabolic processes [58]. Furthermore, kidney weight increased after EN treatment, and RAL had no effect, whereas the combined therapy increased it to the highest level measured among the groups. Testosterone replacement therapy was shown to be able to delay the progression of chronic kidney disease [59]. However, the use of AASs causes significant bile acid nephropathy [60] and is associated with glomerular abnormalities and proteinuria [61]. These side effects of EN and EN + RAL in livers and kidneys were not a part of our study. Therefore, we did not investigate them in detail. However, it is important to report them and conduct further extensive analyses, since EN is marketed as an alternative to ASSs for muscle gain and physical performance, with a superior side-effect profile [57].

The prostate and levator ani are often used as an indicators of the androgenic or anabolic activity of substances [62]. The weights of the levator ani and prostate both increased under EN treatment to different extents. The anabolic effect of EN on the levator ani was stronger (109% of the Non-Orx group) than the androgenic effect on the prostate (51% of the Non-Orx group) (BW-corrected data). Similar observations were published in our previous study [15]. Other preclinical studies also reported a high anabolic activity of EN and only partial androgenic activity [12, 63]. Non-steroidal SARMs exhibit lower androgenic activity than testosterone since they are not aromatized to dihydrotestosterone thus providing promising alternatives for testosterone replacement therapies [12, 64]. RAL alone affected neither the levator ani nor prostate, whereas RAL combined with EN slightly reduced the androgenic effect of EN on prostates (45% of the Non-Orx) and increased its anabolic effect (125% of the Non-Ovx). In our study, prostate weight correlated positively with BW. In the literature, studies report both an association of obesity with prostate volume as well as no correlation of body mass index with prostate volume [65, 66].

Sex hormones play an important role in the regulation of the hypothalamic-pituitary–gonadal (HPG) axis by inhibiting the production of GnRH in the hypothalamus, which stimulates the secretion of LH and FSH by the pituitary. In our study, serum LH level was neither affected by Orx nor the treatments, whereas FSH level was enhanced in the Orx + RAL group. EN was not shown to affect either LH or FSH in preclinical and clinical studies in males [63]. Treating elderly men with RAL was shown to increase serum FSH significantly, with no effect on serum LH [16]. It was suggested that even at low endogenous estradiol levels, RAL continues to function as an estrogen antagonist at the HPG axis [16]. Combining RAL with EN normalized the FSH level in our study, which can be considered an advantage of this treatment.

The present study has several limitations. The study focused on the effect of non-steroidal selective androgen and estrogen receptor modulators applied as a single or combined treatments on bone structure and metabolism, while it lacked direct comparisons with the steroidal hormones as testosterone, testosterone with aromatase inhibitor, dihydrotestosterone and estrogen as well as combinations of these substances. Furthermore, besides analysis of prostate and levator ani weights, seminal vesicle should be weighted in future studies to better understand the effects of the treatments on the male reproductive system and reveal their possible side effects.

In conclusion, both EN and RAL treatments prevented bone loss observed after Orx in rats to some extent. EN possessed mostly an anabolic effect on bone, whereas RAL exhibited antiresorptive activity. An enhanced Mg content in bone, as well as increased liver, kidney, and prostate weights could be possible undesirable side effects of EN treatment. Although there are numerous studies regarding the benefits of EN treatment for musculoskeletal diseases such as osteoporosis and sarcopenia, its recent developments have been directed at therapy for tumor cachexia, stress incontinence, and breast cancer [13, 67]. The favorable effect of RAL on bone and its lack of effect on the prostate can be considered positively in general. However, reductions in BW due to a decreased food intake, as well as the enhancement of FSH, should be taken into consideration when applying this treatment to androgen-deficient male organisms. Though the application of RAL did not show clear beneficial effects on bone tissue in healthy elderly men [16, 68], it might be a therapeutic option for preventing the development of severe osteoporosis in prostate-cancer patients undergoing surgical ablation or pharmacological deprivation of gonadal androgens, as was demonstrated by Smith et al. [17]. The EN + RAL treatment was the most effective in preventing bone loss, combining the osteoprotective properties of both substances. Most of the unfavorable or questionable effects revealed under single therapies were diminished, and bone parameters were normalized to the levels of healthy, Non-Orx rats. Given its observed side effects on liver, kidney, and prostate weight, whether EN + RAL treatment could represent a prevention option for osteoporosis in men should be further investigated.

## Supplementary Information

Below is the link to the electronic supplementary material.

Supplementary file1 (PDF 364 KB)
