# Supplementary material for: A combined treatment with selective androgen and estrogen receptor modulators prevents bone loss in orchiectomized rats
Source: J Endocrinol Invest. 2022 Jul 22;45(12):2299–311. doi: 10.1007/s40618-022-01865-9 (PMC9646546; doi:10.1007/s40618-022-01865-9)
Supplement: Supplementary file 1 — Supplementary file1 (PDF 364 KB) [file 40618_2022_1865_MOESM1_ESM.pdf]

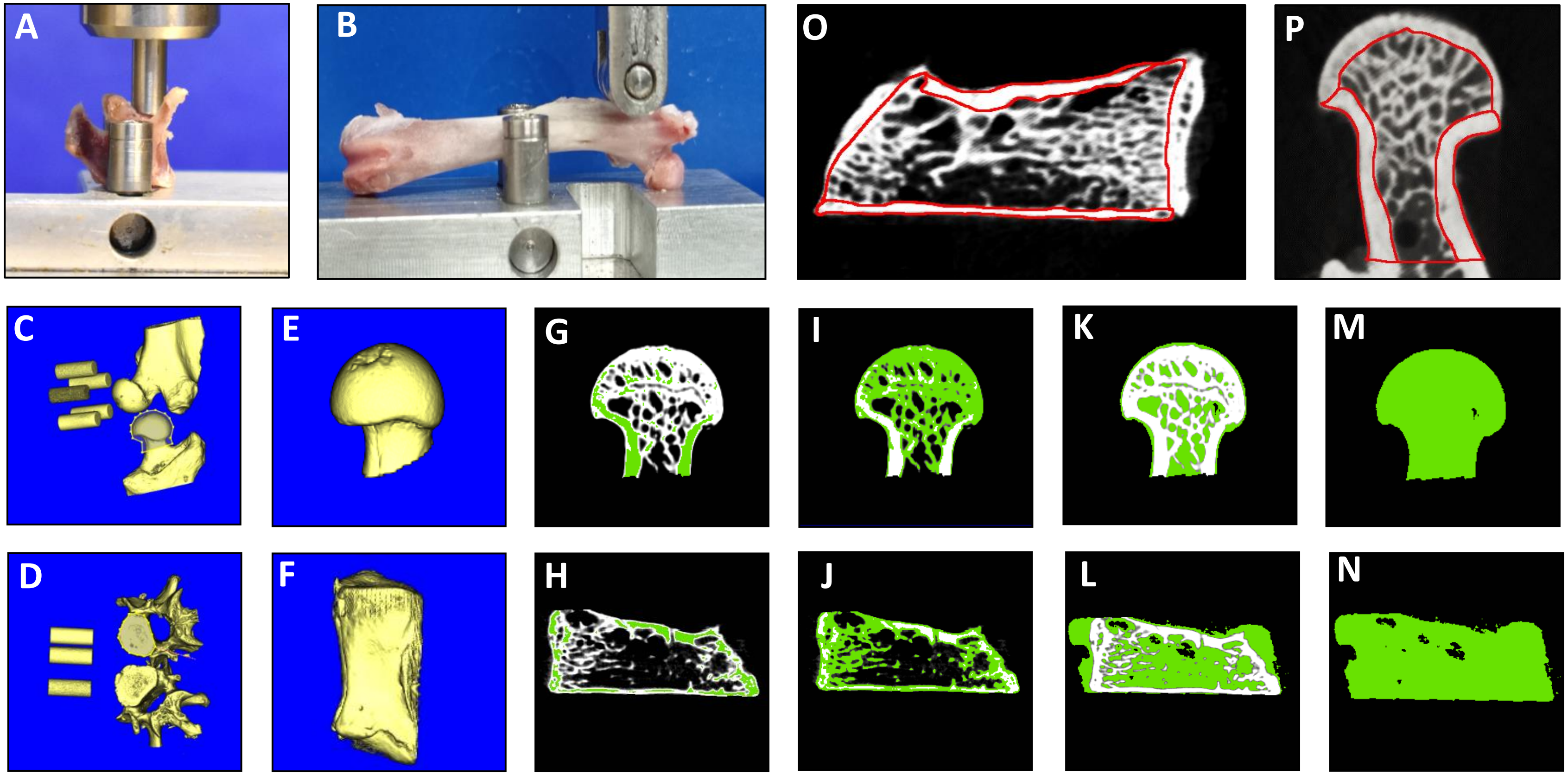

Supplementary Figure 1. (A, B) Biomechanical test of L4 and left femur; (C-N) micro-CT 3D analysis of femora and L4; (O, P) 2D-analysis of L4 and femur images.

(C, D) scans of femur, L4 and 5 phantoms; (E, F) femoral head and corpus vertebra of L4; (G, H) thresholds for cortical bone: femur 1.1 and L4 0.9 g/cm^3 to the highest measured threshold; (I, J) for trabecular bone: femur 0.5 to 1.1 and L4 0.4 to 0.9 g/cm^3; (K, L) for soft tissue: femur -0.1 to 0.5 and L4 -0.1 to 0.4 mg/cm^3; (M, N) for total tissue: femur and L4 -0.1 to the highest threshold; (O, P) red bounded regions: cortical and trabecular bone.
